# Supplementary material for: Timing and co-occurrence of symptoms prior to a diagnosis of light chain (AL) amyloidosis
Source: Blood Cancer J. 2024 May 26;14(1):61. doi: 10.1038/s41408-024-01040-8 (PMC11127981; doi:10.1038/s41408-024-01040-8)

**Supplemental Figure 1**. Estimated probable density of precursor diagnoses by time before AL amyloidosis diagnosis. The distribution of the time between the first diagnosis of each precursor to the AL diagnosis was estimated in the 3-year history cohort using kernel-density estimation. A scaled probit transformation was used to account for the boundary restrictions at -36 months and time 0. The plot shows the period from -2 years to eliminate the “prevalence” period. The dashed vertical line is the median time to precursor within the 2-year incidence period.


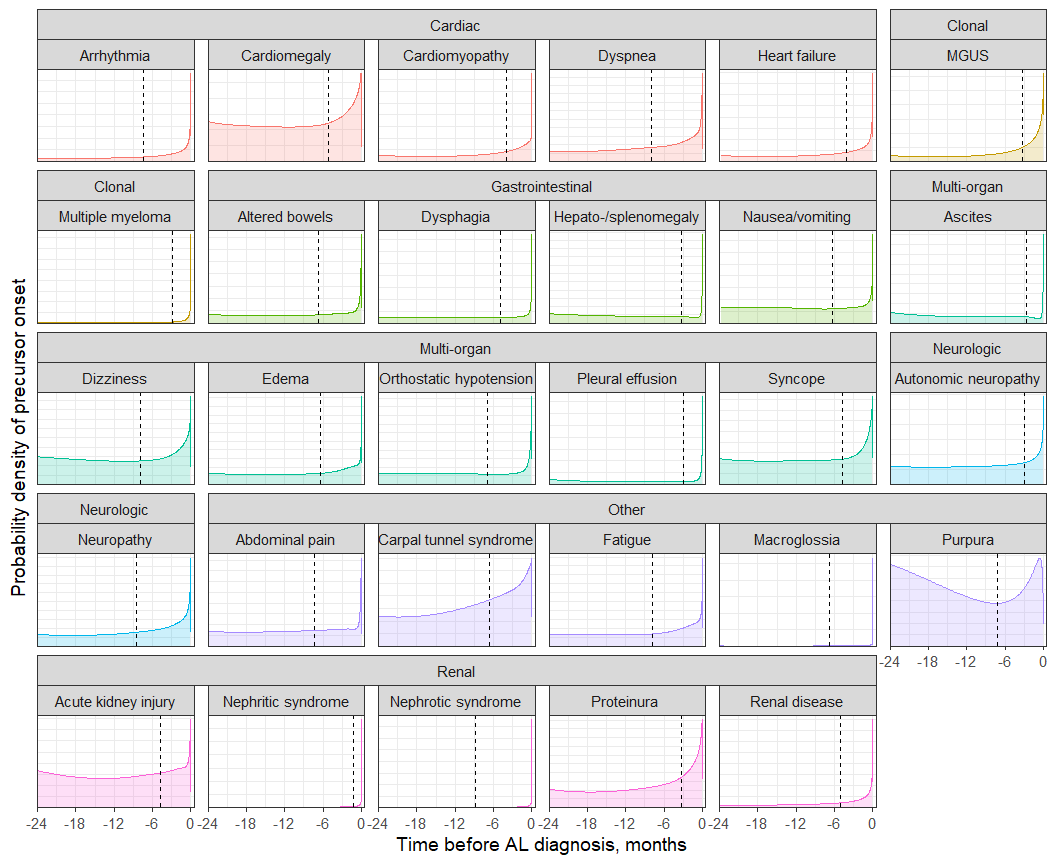


**Supplemental Figure 2**. Estimated probable density of precursor diagnoses by time before AL amyloidosis diagnosis. The distribution of the time between the first diagnosis of each precursor to the AL diagnosis was estimated in the 3-year history cohort using kernel-density estimation. A scaled probit transformation was used to account for the boundary restrictions at -36 months and time 0. The plot shows the period from -6 months to diagnosis.


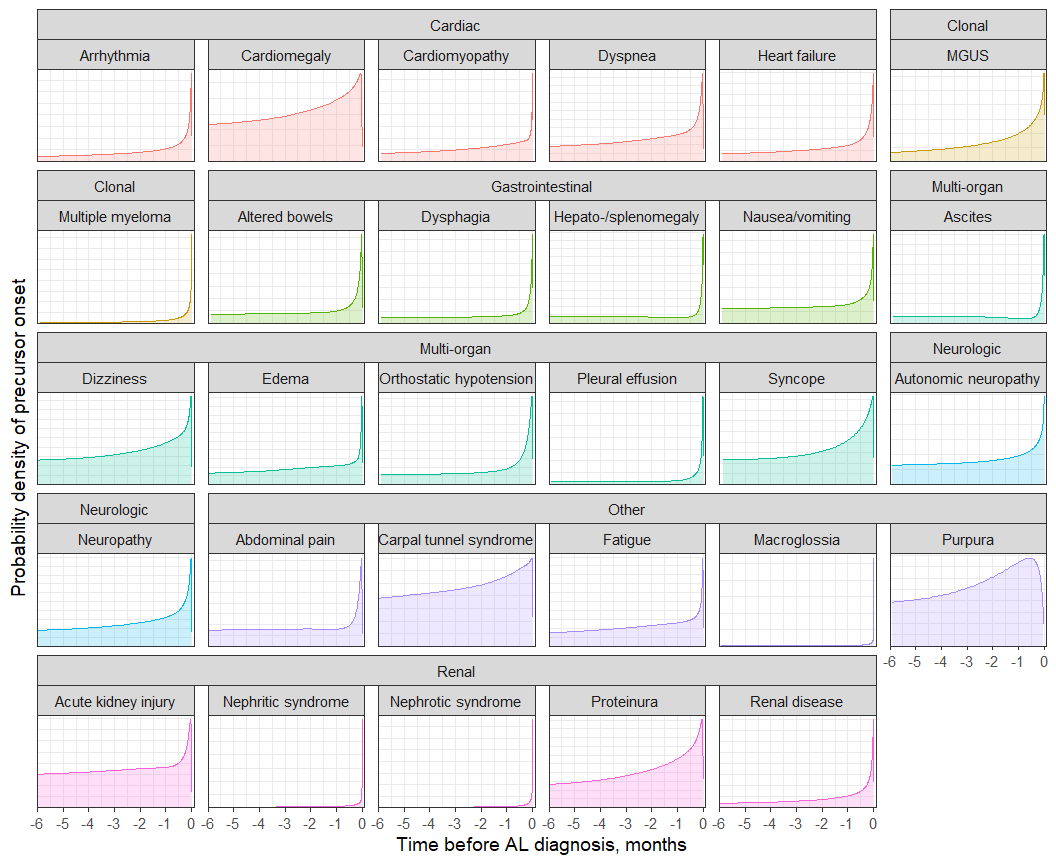

Supplement: Supplementary file 1 — Supplemental material [file 41408_2024_1040_MOESM1_ESM.docx]
